# Impact of cardiac amyloidosis on survival in aortic stenosis patients undergoing TAVR: a systematic review and reconstructed time-to-event meta-analysis

**DOI:** 10.1186/s43044-026-00762-3

**Published:** 2026-06-24

**Authors:** Omar Almaadawy, Naydeen Mostafa, Ahmed Elbataa, Belal Mohamed Hamed, Menna Sarhan, Eman Mohyeldin, Omar Kamel, Amar Asad, Buseyna Dahik, Mohamed Ismael Elnady, Muhammad Ashraf Husain, Ahmed Elshahat, Ahmed Mansour, Christian Eskander, Mustafa Ahmed

**Affiliations:** 1 Department of Cardiovascular Medicine, Saint Louis University, St. Louis, USA; 2 Faculty of Medicine, Ain Shams University, Cairo, Egypt; 3 Faculty of Medicine, Al Azhar University, Cairo, Egypt; 4 Faculty of Medicine, Zagazig University, Zagazig, Egypt; 5 Faculty of Medicine, Menoufia University, Menoufia, Egypt; 6 Faculty of Physical Therapy, South Valley University, Qena, Egypt; 7 Ankara Yıldırım Beyazıt University, Ankara, Turkey; 8 The Wright Center for Graduate Medical Education, Scranton, USA; 9 Department of Medicine, University of Florida, Gainesville, USA; 10 Department of Cardiovascular Medicine, University of Florida, Gainesville, USA

**Keywords:** Aortic stenosis, Cardiac amyloidosis, Transcatheter aortic valve replacement, Survival outcomes, Meta-analysis

## Abstract

**Background:**

New evidence suggests a relatively high prevalence of occult cardiac amyloidosis (CA) among patients with aortic stenosis (AS). While transcatheter aortic valve replacement (TAVR) is an established treatment for AS, the impact of concomitant CA on long-term outcomes remains unclear. We conducted a systematic review and meta-analysis to evaluate survival and procedural outcomes of TAVR in AS patients with and without CA.

**Methods:**

PubMed, Scopus, Web of Science, Google Scholar, and the Cochrane Library were searched through 21 April 2026. Studies comparing outcomes of TAVR in patients with and without CA and reporting Kaplan–Meier survival curves were included. Individual patient survival data were extracted from Kaplan–Meier curves and reconstructed for pooled analysis. Secondary outcomes were analyzed using random-effects meta-analysis.

**Results:**

Seven studies including 2747 patients were analyzed. In the primary analysis, which included both definitive and probability-based definitions of CA, CA was associated with increased all-cause mortality following TAVR (HR: 1.58; 95% CI 1.23, 2.03; *P* < 0.001). However, in a sensitivity analysis restricted to studies with confirmed CA, this association was no longer significant (HR: 1.32, 95% CI 0.84, 2.07, *P* = 0.226). There were no significant differences in pacemaker implantation (OR: 1.33; 95% CI 0.69, 2.56; *P* = 0.40) or more than mild aortic regurgitation (OR: 0.96; 95% CI 0.25, 3.78; *P* = 0.96).

**Conclusion:**

The association between CA mortality after TAVR in AS patients is highly dependent on how CA is defined. While analyses including screening-based cohorts suggest increased risk, this was not observed in analyses of studies with confirmed CA. These findings highlight the impact of differing diagnostic approaches in CA and underscore the need for future studies to use standardized criteria and prospective designs to clarify the independent prognostic role of confirmed CA.

**Supplementary Information:**

The online version contains supplementary material available at 10.1186/s43044-026-00762-3.

## Introduction

Aortic stenosis is the most common clinically significant valvular heart disease in older adults [1]. Its prevalence rises with age, largely due to progressive calcification of the aortic valve [2, 3]. In symptomatic or severe cases, transcatheter aortic valve replacement (TAVR) has been shown to be an effective treatment, improving symptoms, quality of life, and clinical outcomes [4]. However, a substantial proportion of patients referred for TAVR have coexisting cardiac amyloidosis (CA), which is frequently underrecognized in routine clinical practice [5]. In this population, CA is most commonly related to transthyretin amyloid deposition and results in a restrictive cardiomyopathy that adversely affects myocardial structure and function [6, 7]. As both AS and CA are age-associated conditions, their coexistence is increasingly encountered in contemporary TAVR populations.

According to recent studies, up to 16% of older adults with severe AS undergoing TAVR may also have CA [8, 9]. This overlap has important clinical implications, as myocardial amyloid infiltration may complicate diagnostic evaluation and contribute to persistent functional limitation despite correction of valvular obstruction [10]. Consequently, the presence of CA may attenuate the symptomatic and prognostic benefit typically expected after TAVR in patients with isolated AS.

It is important to recognize CA in patients undergoing TAVR, as evidence shows it may impact procedures’ success and long-term outcomes [11, 12]. Studies indicate that patients with both AS and CA may experience less improvement after TAVR compared to those with AS alone, possibly because the restrictive effects of amyloid deposits persist despite valve replacement [13]. Unfortunately, many cases of CA continue to be underdiagnosed in this population, partly due to the limited use of diagnostic tools specialized to detect amyloidosis. However, multimodality imaging methods, such as nuclear scintigraphy with bone-seeking tracers and strain imaging, are now proving useful in non-invasively identifying CA, providing valuable insights for better patient management [14].

Despite growing recognition of CA coexistence, the impact of CA on clinical outcomes following TAVR remains incompletely defined. Individual studies have reported heterogeneous results, and uncertainty persists regarding how the presence of CA modifies survival or procedural outcomes after valve intervention. A comprehensive synthesis of available evidence is therefore needed to clarify the prognostic implications of concomitant CA in patients undergoing TAVR and to inform management strategies in this high-risk population. This systematic review and reconstructed time-to-event meta-analysis aims to determine whether the presence of CA, as identified in real-world clinical practice, is associated with differences in survival and procedural outcomes following TAVR in patients with AS.

## Methods

### Study design and search strategy

This study was conducted according to the Preferred Reporting Items for Systematic Reviews and Meta-Analyses (PRISMA) guidelines. Ethical review for this study was waived as this study used de-identified, publicly available data. The protocol for this study was registered on the International Prospective Register of Systematic Reviews (CRD42024606453).

We searched PubMed, Scopus, Web of Science, Google Scholar, and Cochrane CENTRAL for studies published up to 12 October 2024. A search string of relevant keywords was employed: (Transcatheter aortic valve replacement OR TAVR OR TAVI OR Aortic valve replacement) AND (amyloid* OR AA OR ATTR OR AL) AND (outcome* OR prognosis OR mortality OR risk* OR risk stratification OR adverse event*). Two authors independently screened the results by title and abstract, then by full-text. Any conflicts were resolved by a third author. *We used Covidence* software (*Veritas Health Innovation*,* Melbourne*,* Australia*; http://www.covidence.org) to streamline the study selection process. The search was repeated on 21 April 2026 prior to the final results to ensure that no newly published studies were missed.

### Eligibility criteria

Studies were considered for inclusion if (1) they were written in English, (2) The population comprised patients who underwent TAVR, (3) there was a direct two-arm comparison between with CA and AS patients without CA, (4) Outcomes reported included survival or all-cause mortality displayed in a Kaplan-Meier (KM) curve. Studies combining surgical and transcatheter management in the same aortic valve intervention group were excluded. Case reports, case series, commentaries, editorials, expert opinions, conference presentations, reviews, and animal studies were excluded. Both randomized controlled trials and observational studies were eligible for inclusion. No restrictions were applied on the publication date. Eligible studies from the same institution or by the same contributing authors were cross-examined and excluded if the risk of double counting could not be eliminated.

### Risk of bias assessment

Two authors independently performed quality assessment of included studies using the Newcastle-Ottawa Scale (NOS). Conflicts in assessment were resolved by consulting a third author. Studies with a score of seven or more were considered to be high-quality studies with low risk of bias.

### Data extraction

Two authors independently extracted data from selected studies. Discrepancies were resolved by consulting a third author. Data extracted included study design, country, sample size, CA method of diagnosis, follow-up duration, demographic characteristics, baseline echocardiographic parameters of patients, and reported outcomes. When key data were unavailable in the published reports or supplementary materials, attempts were made to contact the corresponding authors for clarification.

### Data analysis

For our primary outcome, a two-stage approach was implemented. First, we utilized the web-based program *WebPlotDigitizer* to digitize the KM curve from each included study. Raw data coordinates – specifically, time and survival probability – were extracted from the two respective groups on the curve. Thereafter, data coordinates were processed based on the extracted raw coordinates and, if available, the numbers at risk at given time points to reconstruct individual patient data (IPD). Numbers-at-risk tables were available for six of the seven included studies and were incorporated into the reconstruction process. For the remaining study, IPD reconstruction was based on the digitized KM coordinates and available published survival information. For meta-analysis of aggregate time-to-event data, IPD data from all included studies were merged into one final dataset. Finally, this pooled data was visualized in a KM curve. Analyses included a primary full-cohort analysis incorporating all eligible studies, including one study that classified CA based on an artificial intelligence (AI) enabled electrocardiogram (ECG) probability model, and a restricted sensitivity analysis, not specified in the protocol, limited to studies using definitive diagnostic criteria for CA to explore potential sources of heterogeneity and assess the robustness of findings. Statistical analysis was conducted on R Statistical Software (*Foundation for Statistical Computing*,* Vienna*,* Austria*) (version 4.3.1) and utilized the R packages “IPDfromKM” (version 0.1.10), “survival” (version 3.7.0), and “survminer” (version 0.5.0).

Hazard ratio (HR) with 95% confidence intervals (CI), were calculated using a one-stage Cox proportional hazards model stratified by study to account for clustering of reconstructed individual patient data within the original studies. To assess the robustness of the findings, shared frailty Cox, mixed-effects Cox, and two-stage random-effects meta-analytic models were also performed. The proportional hazards assumption was assessed using Schoenfeld residuals. Additionally, we assessed postoperative outcomes of interest in included studies, which were more than mild aortic regurgitation and pacemaker implantation. Effect estimates were pooled in a random-effects two-arm meta-analysis to calculate odds ratio (OR) and 95% CI. A two-sided P-value of < 0.05 was considered statistically significant. The I^2^ statistics and Cochrane Q test were used to analyze statistical heterogeneity. A value of ≥ 50% in the I^2^ statistic or a P-value of < 0.10 on the Cochrane Q test was considered as significant heterogeneity between the studies [15, 16].

## Results

### Literature search

**Fig. 1:**
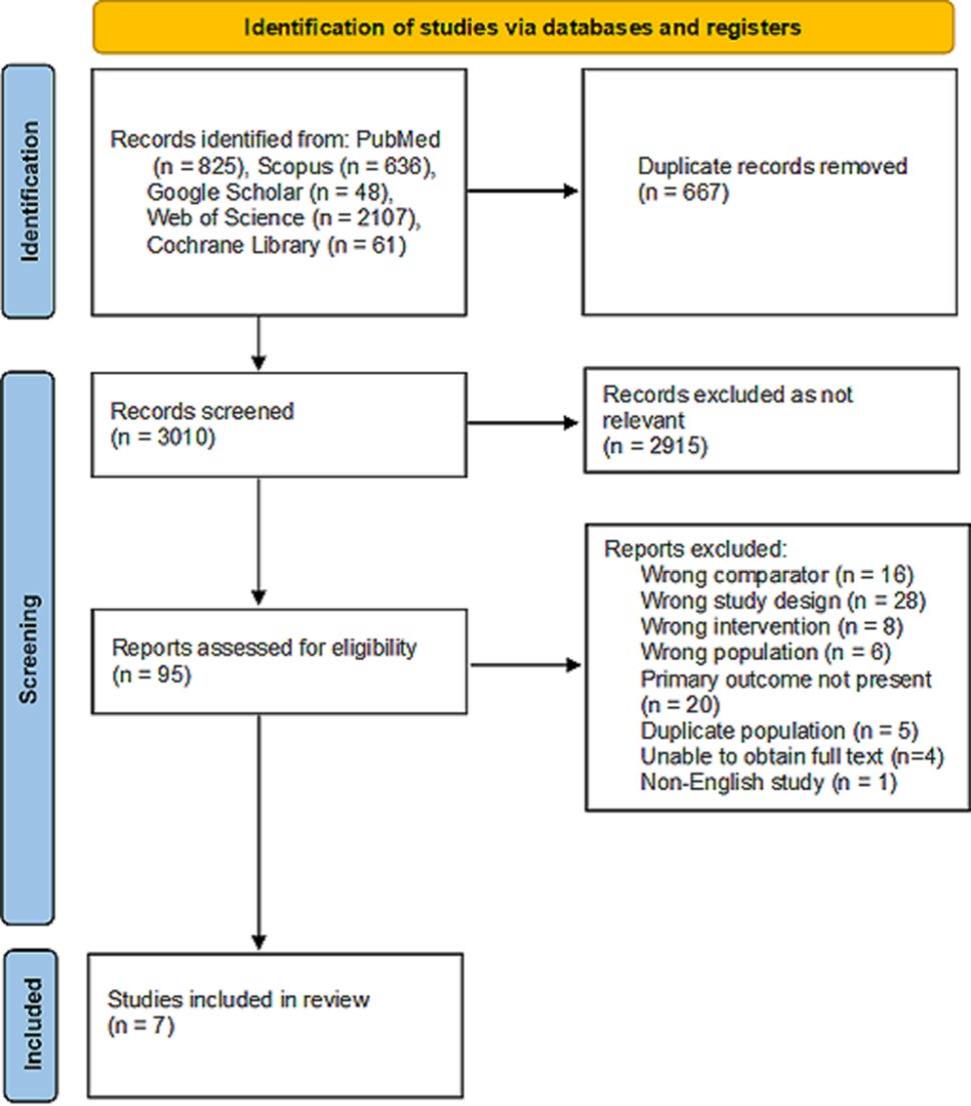
PRISMA flowchart for study selection

The PRISMA flowchart, as shown in Fig. 1, outlines the workflow of identifying relevant studies. There were initially 3010 studies identified through database searches before duplicate removal. Ultimately, seven studies met the inclusion criteria [10, 17–22].

## Study characteristics

The characteristics of the included studies are displayed in Table 1. In total, 2848 patients (2383 AS patients without CA and 465 AS patients with CA) were included. All included studies had a cohort design and followed patients either retrospectively or prospectively. Of the seven included studies, three were conducted in the United States of America, one was conducted in France, one was conducted in Germany, one was conducted in Switzerland, and one was conducted in England. The baseline demographic characteristics and hemodynamic parameters of the included patients in the meta-analysis are displayed in Table 2.

**Table 1:** Characteristics of included studies

Source	Design	Country	Method of CA diagnosis	Quality by NOS	Median follow-up duration (months)
Scully et al. 2020 [10]	Prospective cohort	England	DPD bone scintigraphy	High quality	19
Rosenblum et al. 2021 [20]	Prospective cohort	USA	Tc99m-PYP cardiac scintigraphy	High quality	24
Costa et al. 2024 [17]	Prospective cohort	France	Bone scintigraphy	High quality	12
Beuthner et al. 2024 [21]	Prospective cohort	Germany	Histological assessment	High quality	28
Pietri et al. 2024 [19]	Retrospective cohort	USA	Electrocardiogram artificial intelligence algorithm	High quality	12
Dobner et al. 2023 [18]	Prospective cohort	Switzerland	DPD bone scintigraphy	High quality	12
Maenza et al. 2025 [22]	Retrospective cohort	USA	Tc99m-PYP cardiac scintigraphy or histological assessment	High quality	17.8

NOS: Newcastle Ottawa Scale; CA: cardiac amyloidosis; DPD: 99mTc-3,3-diphosphono-1,2-propanodicarboxylic acid scintigraphy; Tc99m-PYP: technetium-99 m pyrophosphate; USA: United States of America

**Table 2:** Baseline demographic and hemodynamic characteristics of full cohort

Source	Overall sample size (CA-/CA+)	Age (CA-/CA+)	No. of males (CA-/CA+)	DM (CA-/CA+)	HTN (CA-/CA+)	LVEF (CA-/CA+)	Aortic valve area, cm^2^ (CA-/CA+)	Aortic valve mean gradient, mm Hg (CA-/CA+)
Scully et al. 2020 [10]	200 (174/26)	85.0 (85.0/88.0)	99 (83/16)	48 (45/3)	154 (135/19)	54 (54/54)	0.73 (0.73/0.74)	41.0 (42.0/37.0)
Rosenblum et al. 2021 [20]	204 (177/27)	83.0 (82.0/86.0)	133 (107/26)	NR	175 (150/25)	54 (55/48)	0.76 (0.76/0.80)	40.0 (41.0/35.0)
Costa et al. 2024 [17]	100 (93/7)	82.0 (81.7/85.0)	60 (54/6)	35 (32/3)	84 (77/7)	59.0 (59.0/60.0)	0.76 (0.75/0.85)	46.7 (47.0/43.7)
Beuthner et al. 2024 [21]	162 (154/8)	79.8 (79.8/82.4)	105 (98/7)	65 (62/3)	145 (137/8)	51.9 (51.9/51.22)	0.7 (0.7/0.8)	35.93 (36.10/23.0)
Pietri et al. 2024 [19]	1426 (1077/349)	81.0 (81.2/80.5)	821 (577/244)	481 (345/136)	1195 (887/308)	57.4 (58.9/53)	NR	NR
Dobner et al. 2023 [18]	315 (281/34)	83.1 (82.8/85.7)	190 (162/28)	95 (88/7)	278 (246/32)	58.0 (58.7/52.8)	0.7 (0.7/0.8)	36.0 (37.3/25.4)
Maenza et al. 2025 [22]	441 (427/14)	81.2 (81/86)	279 (267/12)	180 (176/4)	408 (395/13)	NR	NR	NR

CA: cardiac amyloidosis; DM: diabetes mellitus; HTN: hypertension; LVEF: left ventricular ejection fraction; NR: not reported

### Primary survival analysis: full cohort

**Fig. 2:**
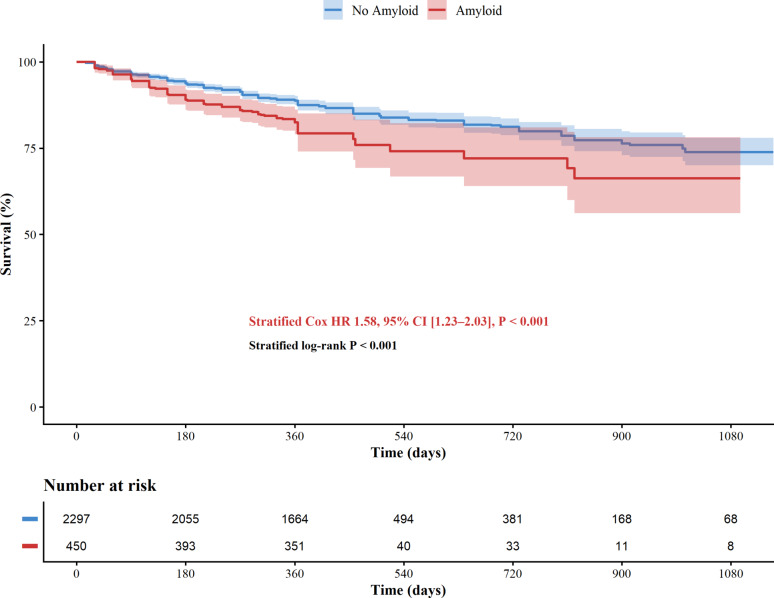
Pooled reconstructed Kaplan-Meier curve for long-term survival after TAVR, including the full patient cohort

The primary time-to-event meta-analysis, which incorporated studies that stratified patients using either a definitive diagnostic method or a probability-based classification (high vs. low probability of CA), included 2,747 of the 2,848 patients from seven studies, corresponding to those represented in the published KM curves. Following TAVR, AS patients classified as having CA had a significantly higher risk of all-cause mortality compared with AS patients classified as not having CA (HR: 1.58; 95% CI 1.23, 2.03; *P* < 0.001) (Fig. 2). Sensitivity analyses using shared frailty Cox, mixed-effects Cox, and two-stage random-effects meta-analytic models yielded highly consistent effect estimates (Supplemental Table 1). The proportional hazards assumption was satisfied based on Schoenfeld residuals (global test *P* = 0.61). The restricted mean survival time was lower in AS patients classified as having CA (28.0 months vs. 30.5 months), with a median follow-up duration ranging across studies from 12 months to 28 months.

### Sensitivity analysis: restricted cohort

**Fig. 3:**
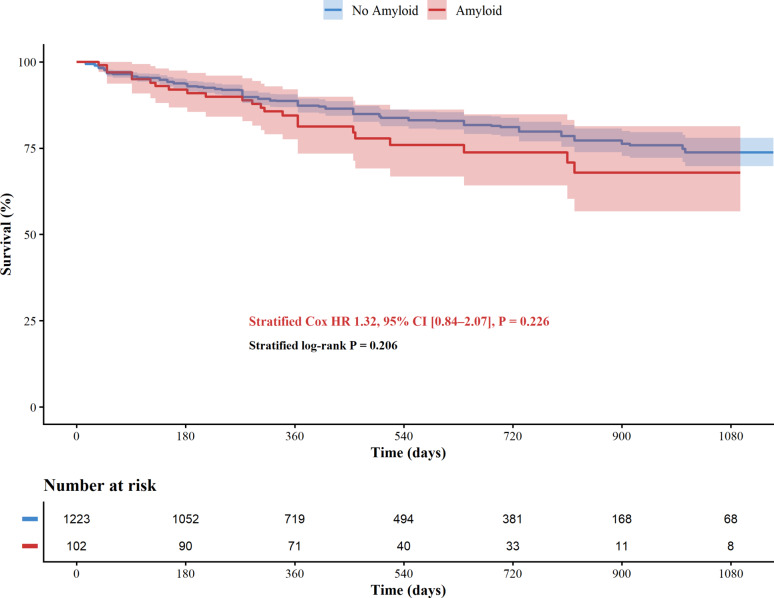
Pooled reconstructed Kaplan-Meier curve for long-term survival after TAVR, including only patients with confirmed CA diagnosis

Our secondary time-to-event meta-analysis, limited to studies with definitive diagnostic methods, included 1325 patients from six studies. In contrast to the primary analysis, there was no significant difference in the risk of all-cause mortality between AS patients with CA and those without CA following TAVR (HR = 1.32, 95% CI 0.84, 2.07, *P* = 0.226) (Fig. 3). Sensitivity analyses using shared frailty Cox, mixed-effects Cox, and two-stage random-effects meta-analytic models yielded highly consistent effect estimates (Supplemental Table 1). The proportional hazards assumption was satisfied based on Schoenfeld residuals (*P* = 0.72). The restricted mean survival time was lower in AS patients classified as having CA (28.6 months vs. 30.5 month), with a median follow-up duration also ranging across studies from 12 months to 28 months.

### Secondary outcomes

**Fig. 4:**
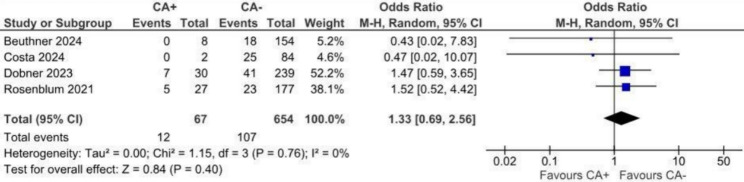
Forest plot for comparing new pacemaker implantation after TAVR

**Fig. 5:**
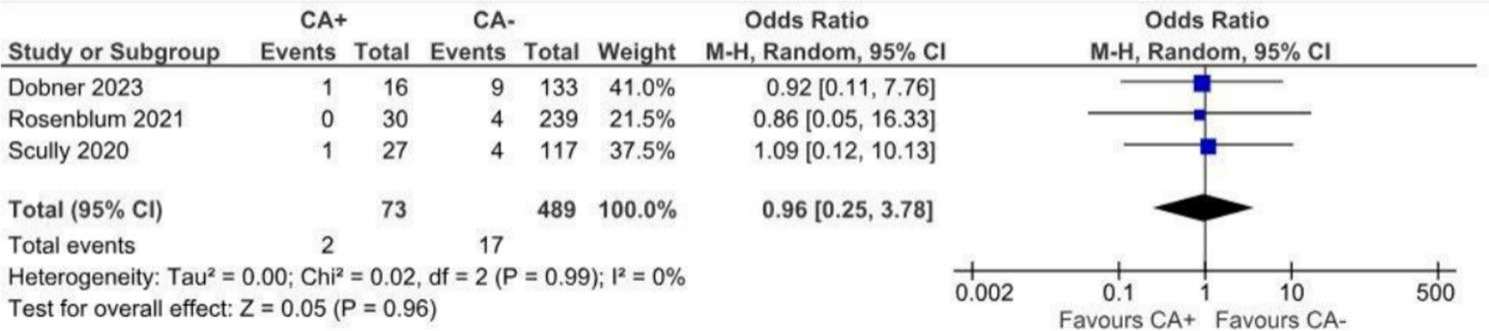
Forest plot for comparing aortic regurgitation after TAVR

Four studies reported the incidence of new pacemaker implantation following TAVR [17, 18, 20, 21]. The pooled odds ratio showed no significant difference between AS patients with CA and those without CA (OR: 1.33; 95% CI 0.69, 2.56; *P* = 0.40) (Fig. 4). There was no significant heterogeneity between the studies (I^2^ = 0%; *P* = 0.76). Three studies [10, 18, 20] reported the incidence of more than mild aortic regurgitation following TAVR. The pooled OR showed no statistically significant difference between AS patients with CA and those without CA (OR: 0.96; 95% CI 0.25, 3.78; *P* = 0.96) (Fig. 5). There was no significant heterogeneity between the studies (I^2^ = 0%; *P* = 0.99).

## Discussion

This study provides the first reconstructed time-to-event meta-analysis to evaluate survival following TAVR in AS patients with versus without CA, as well as a meta-analysis of secondary procedural outcomes. We conducted two separate reconstructions: a primary analysis that incorporated studies using both definitive and probability-based CA classification, and a restricted analysis that included only studies with definitive diagnostic methods and excluded the Pietri et al. study, which identified CA using an AI-based ECG model to classify patients by probability of CA. In the primary analysis, AS patients classified as having CA had a significantly higher risk of all-cause mortality compared with AS patients classified as not having CA. In the restricted analysis, including fewer patients and only studies with a confirmed CA diagnosis (using scintigraphy or histology), no significant survival difference was detected.

The discrepancy between the HR in the primary analysis versus the restricted analysis shows that the prognosis of CA patients depends largely on how the CA population is defined. Our primary analysis included a large cohort from the study by Pietri et al. comprising 1426 of the 2747 patients included in the pooled cohort (> 50% of the total study population), making it the dominant contributor to the primary analysis [19]. As acknowledged by the authors of the study, the AI-based ECG models used are designed for screening and risk stratification rather than definitive diagnosis. Hence, the population identified in Pietri et al. likely represented a broader CA cohort, potentially including patients with subclinical, rather than a cohort directly comparable to those with confirmed myocardial amyloid deposition. However, because these AI algorithms are intended as risk-stratification, the identified population may also include patients with other forms of advanced structural heart disease, myocardial remodeling, fibrosis, or conduction abnormalities that share electrocardiographic features with CA and are independently associated with adverse outcomes. Upon exclusion of this study, no survival difference was found between the groups.

The different diagnostic approaches used by the included studies in our analysis highlight the clinical reality of CA diagnosis. Currently, the diagnostic criteria for CA encompasses both invasive and non-invasive techniques. The invasive criteria, which historically were the only method for disease confirmation, are tissue biopsy [23]. Recent advances in scintigraphy enabled the development of non-invasive criteria [24]. This evolution in diagnostics is reflected in a recent analysis of U.S. mortality data using the national U.S. mortality database, which showed a marked increase in CA-related mortality on death certificates, despite advancements in medical and surgical management of CA [25]. This temporal pattern was largely attributed to the changes in diagnostic pathways for CA, rather than a true increase in disease incidence alone, and suggests that changes in methods of diagnosis can meaningfully alter the type and size of the population captured under the label of CA.

Nevertheless, CA patients are still commonly diagnosed in the later stages of the disease course, when treatment options are limited and prognosis is poor [26, 27]. In a prospective analysis of patients with wild-type transthyretin CA, a diagnostic delay of over 4 years was observed in 42% of patients [28]. A targeted review with 23 included studies reported a mean diagnostic delay of 6.1 years for wild-type CA and 5.7 years for hereditary CA [29]. This puts into perspective the surge in recent literature evaluating and testing the use of AI in CA diagnosis, which is predicted to enable earlier and more consistent recognition of disease signals that are often subtle and distributed across multiple diagnostic modalities [30–32]. Recently, Grogan et al. developed an AI tool that predicted CA more than six months before clinical diagnosis in over half of patients [33]. However, these approaches are primarily designed for risk stratification and screening before further confirmatory testing.

Hence, the discrepancy between our primary and restricted analyses should be interpreted with caution as it reflects the evolving and heterogeneous nature of CA diagnosis in contemporary clinical practice, where the diagnostic landscape has expanded significantly, moving from endomyocardial biopsy to scintigraphy and, most recently, to AI-assisted screening tools. Each of these approaches captures a distinct point along the diagnostic spectrum. AI-ECG models identify a broader cohort with poorer prognosis and a higher likelihood of undiagnosed CA [19].

Potential pathophysiological mechanisms may help explain the higher mortality observed in these certain CA cohorts following TAVR. The accumulation of amyloid fibrils can involve multiple cardiovascular structures, including the valves and myocardium, sometimes extending from the base to the apex of the left ventricle, and leading to clinical symptoms of heart failure, arrhythmias, and cardioembolic events [34, 35]. Importantly, the presence of pre-procedural advanced cardiac damage is associated with a substantially increased risk of all-cause mortality in patients who have undergone TAVR, with a study reporting up to a fourfold increase in risk [36].

Systemic effects of amyloidosis may also contribute to the lower survival observed after TAVR in AS patients with concomitant CA. For instance, Beuthner et al. who conducted a prospective single-center study of 162 AS patients with and without CA, found higher rates of acute kidney injury (AKI) in those with CA compared with those without [21]. They also observed higher rates of baseline renal impairment in AS patients with concomitant CA, which may predispose this subgroup to a higher incidence of AKI. These higher AKI rates may reflect systemic amyloid involvement of the kidneys or renal ischemic injury, both of which could plausibly contribute to adverse post-procedural outcomes [37, 38].

Beyond survival and symptomatic benefit, procedural safety and device-related complications are also important considerations when evaluating the role of TAVR in the management of CA [39–41]. Conduction disturbances requiring permanent pacemaker implantation are among the most common complications following TAVR [42]. However, our analysis showed no significant difference in pacemaker implantation rates between patients with and without CA. This finding may reflect that conduction disturbances after TAVR are largely influenced by procedural factors, including valve type, implantation depth, and mechanical compression of the conduction system, which are common to both groups, potentially attenuating any additional risk conferred by amyloid infiltration [43].

Additionally, our study found no significant difference between the two groups and more than mild aortic regurgitation rates. Aortic regurgitation after TAVR typically occurs when the prosthetic valve does not completely seal against the native valve annulus [44]. This may result from annular–prosthesis size or shape mismatch, severe native valve calcification, incomplete prosthesis expansion, or suboptimal prosthesis positioning [44–46]. Taken together, these findings suggest that the presence of concomitant CA does not appear to confer excess procedural risk with respect to major device-related complications following TAVR.

## Limitations

The results of our study should be interpreted within the context of its limitations. A key limitation of this study is the heterogeneity in the definition of CA across included studies. Specifically, CA was defined using histology, scintigraphy, or an AI-based ECG model, which represent fundamentally different constructs and are not directly interchangeable. Importantly, the risk of misclassification bias in the primary analysis, with the inclusion of the Pietri et al. study, cannot be excluded. Moreover, the shift observed in our KM curves after removing Pietri et al.’s study raises uncertainty as to whether CA has no independent effect on post-TAVR survival, or whether the reduced sample size of the remaining studies limited statistical power to detect a difference. Additionally, our KM curves for CA patients showed a wider CI, especially later in the follow-up period, which reflects greater uncertainty, particularly due to smaller numbers at risk later in follow-up. Further, hemodynamic parameters, including aortic valve area and mean transvalvular gradient, were not consistently reported in included studies, which limits baseline comparability of AS severity across study cohorts. Moreover, most included studies were observational, which introduces the potential for selection bias. In addition, information regarding disease-modifying therapies for CA, including transthyretin stabilizers such as Tafamidis, was not reported in our included studies and therefore could represent a potential source of unmeasured confounding. This limitation is particularly relevant given the increasing availability and adoption of Tafamidis during the study period. Temporal and geographic differences in access to and utilization of disease-modifying therapies may have influenced survival outcomes independently of TAVR and could have contributed to differences in outcomes. Although a randomized controlled trial comparing TAVR with optimal medical therapy in AS patients with and without CA would provide the highest level of evidence, such a design is unlikely to be feasible given ethical and practical considerations.

## Conclusion

All in all, the restricted analysis, limited to studies with definitive diagnostic criteria, provides the most conservative estimate of the independent prognostic impact of confirmed CA following TAVR. The primary analysis, which incorporates the larger AI-derived cohort, reflects the broader population increasingly encountered in real-world practice, when dedicated amyloid centers and scintigraphy are not universally available. Nonetheless, similar rates of pacemaker implantation and more than mild aortic regurgitation were observed between the two groups. Future research should prioritize standardized diagnostic definitions to improve cross-study comparability, while recognizing that restricting evidence exclusively to biopsy-confirmed cohorts would systematically exclude the majority of patients identified through modern diagnostic pathways.

## Supplementary Information

Below is the link to the electronic supplementary material.

Supplementary Material 1.

Supplementary Material 2.

## Data Availability

The datasets analysed during the current study are available from the corresponding author on reasonable request.

## Abbreviations

AF: Atrial fibrillation
AI: Artificial Intelligence
AKI: Acute kidney injury
AS: Aortic stenosis
CA: Cardiac amyloidosis
KM: Kaplan-Meier
NOS: Newcastle-Ottawa Scale
PRISMA: Preferred Reporting Items for Systematic Reviews and Meta-Analyses
TAVR: Transcatheter aortic valve replacement
