# Supplementary material for: Impact of cardiac amyloidosis on survival in aortic stenosis patients undergoing TAVR: a systematic review and reconstructed time-to-event meta-analysis
Source: Egypt Heart J. 2026 Jun 24;78:49. doi: 10.1186/s43044-026-00762-3 (PMC13294412; doi:10.1186/s43044-026-00762-3)
Supplement: Supplementary file 2 — Supplementary Material 2. [file 43044_2026_762_MOESM2_ESM.docx]

Supplemental Table 1. Comparison of hazard ratio estimates across alternative approaches accounting for study-level clustering and heterogeneity

| **Analysis** | **Model** | **HR (95% CI)** | **P value** |
| --- | --- | --- | --- |
| Full cohort | Naive Cox | 1.59 (1.25 to 2.02) | < 0.001 |
|  | Stratified Cox | 1.58 (1.23 to 2.03) | < 0.001 |
|  | Shared frailty Cox | 1.60 (1.24 to 2.05) | < 0.001 |
|  | Mixed effects Cox | 1.60 (1.24 to 2.05) | < 0.001 |
|  | Random effects meta-analysis | 1.58 (1.09 to 2.30) | 0.016 |
| * Restricted cohort | Naive Cox | 1.38 (0.89 to 2.12) | 0.146 |
|  | Stratified Cox | 1.32 (0.84 to 2.07) | 0.226 |
|  | Shared frailty Cox | 1.39 (0.90 to 2.16) | 0.141 |
|  | Mixed effects Cox | 1.39 (0.90 to 2.16) | 0.141 |
|  | Random effects meta-analysis | 1.43 (0.79 to 2.60) | 0.240 |

* Restricted cohort excludes Pietri et al.
